# Caspase-7: a critical mediator of optic nerve injury-induced retinal ganglion cell death

**DOI:** 10.1186/s13024-015-0039-2

**Published:** 2015-08-26

**Authors:** Shreyasi Choudhury, Yang Liu, Abbot F. Clark, Iok-Hou Pang

**Affiliations:** North Texas Eye Research Institute, University of North Texas Health Science Center, 3500 Camp Bowie Blvd, Fort Worth, TX 76107 USA; Pharmaceutical Sciences, University of North Texas Health Science Center, Fort Worth, TX USA; Cell Biology & Immunology, University of North Texas Health Science Center, Fort Worth, TX USA

**Keywords:** Caspase-7, Optic nerve injury, Retinal ganglion cell, Neuroprotection

## Abstract

**Background:**

Axonal injury of the optic nerve (ON) is involved in various ocular diseases, such as glaucoma and traumatic optic neuropathy, which leads to apoptotic death of retinal ganglion cells (RGCs) and loss of vision. Caspases have been implicated in RGC pathogenesis. However, the role of caspase-7, a functionally unique caspase, in ON injury and RGC apoptosis has not been reported previously. The purpose of this study is to evaluate the role of caspase-7 in ON injury-induced RGC apoptosis.

**Results:**

C57BL/6 (wildtype, WT) and caspase-7 knockout (*Casp7*^*−/−*^) mice were used. We show that ON crush activated caspase-7 and calpain-1, an upstream activator of caspase-7, in mouse RGCs, as well as hydrolysis of kinectin and co-chaperone P23, specific substrates of caspase-7. ON crush caused a progressive loss of RGCs to 28 days after injury. Knockout of caspase-7 partially and significantly protected against the ON injury-induced RGC loss; RGC density at 28 days post ON crush in *Casp7*^*−/−*^ mice was approximately twice of that in WT ON injured retinas. Consistent with changes in RGC counts, spectral-domain optical coherence tomography analysis revealed that ON crush significantly reduced the *in vivo* thickness of the ganglion cell complex layer (including ganglion cell layer, nerve fiber layer, and inner plexiform layer) in the retina. The ON crush-induced thinning of retinal layer was significantly ameliorated in *Casp7*^*−/−*^ mice when compared to WT mice. Moreover, electroretinography analysis demonstrated a decline in the positive component of scotopic threshold response amplitude in ON crushed eyes of the WT mice, whereas this RGC functional response was significantly higher in *Casp7*^*−/−*^ mice at 28 days post injury.

**Conclusion:**

Altogether, our findings indicate that caspase-7 plays a critical role in ON injury-induced RGC death, and inhibition of caspase-7 activity may be a novel therapeutic strategy for glaucoma and other neurodegenerative diseases of the retina.

**Electronic supplementary material:**

The online version of this article (doi:10.1186/s13024-015-0039-2) contains supplementary material, which is available to authorized users.

## Background

Axonal injuries occur in a wide range of CNS disorders. They contribute to neuronal damage in diseases and trauma of the human brain and spinal cord. Similarly, injury to the optic nerve (ON), an extension of the CNS, is associated with various ocular diseases and abnormalities, such as glaucoma, traumatic optic neuropathy, ischemic optic neuropathy, and compressive optic neuropathy. In humans, there are approximately 1.2 million axons in the ON. They originate from retinal ganglion cells (RGCs) in the inner retina and are responsible in transmitting image-forming visual and other signals from the retina to the visual centers and circadian pacemakers in the brain [1]. Damage to these axons leads to RGC apoptotic death, and consequently, loss of vision and disturbance of the circadian rhythm [2].

Apoptosis of RGC involves caspases. Caspases are a family of cysteine proteases that activate apoptotic pathways. They serve as either ‘initiators’ or ‘effectors’ based on their molecular functions. The initiator caspases, such as caspases-2, −8, −9, and −10, cleave and activate effector caspases, such as, caspases-3, −6, and −7, which, in turn, hydrolyze target proteins and initiate apoptosis [3]. The involvement of specific caspases in optic neuropathy, glaucoma, and RGC death has been previously implicated. For example, in the ON of glaucoma patients, more axons were found to express caspase-3 [4]. Similarly, activation of specific caspases occurs in retinas of animal models of ON injury, glaucoma, and RGC degeneration: (a) ON crush activates caspase-2 [5]; (b) ON axotomy activates caspases-3, −6, −8, and −9 in RGCs [6–11]; (c) higher levels of active caspases-3, −8, and −9 were shown in the rat retina subjected to chronic ocular hypertension [12–17]; (d) retinal ischemia/reperfusion activates both caspases-2 and −3 [18, 19] with a reported reduction in caspase-7 expression [19]; and (e) intravitreal injection of kainate cleaved caspase-3 with an increase in effector caspase activity [20]. Interestingly, other than caspase-2 [5, 21], inhibition or down-regulation of other initiator caspases or caspase-3 only provides partial protection against injury-induced RGC death [7–9, 12, 16].

Despite the studies described above, very little is known about the involvement of a unique caspase, caspase-7, in RGC apoptosis. Until recently, contribution of caspase-7 to neuronal apoptosis was controversial. Previously, it was generally believed that caspase-7 was not present in the CNS, not activated if present, or even if activated, ineffective in inducing apoptosis [22]. Furthermore, because caspase-7 exhibits a high degree of homology with caspase-3, it was speculated that caspase-7 simply served as a redundant version of caspase-3, and thus did not play a critical role in the apoptosis cascade [22]. However, it was later determined that caspase-7 cleaves exclusive substrates different from those of caspase-3, such as kinectin and co-chaperone P23 [23–25]. Several other non-ocular studies strongly suggest that caspase-7 has a critical, non-redundant role in apoptotic cell death and in normal development [26, 27]. For example, Larner et al. demonstrated that caspase-7 is upregulated and activated in the rat brain after traumatic brain injury [28]. Unlike other caspases, only caspase-7 can be activated by calpain-1 [29], indicating that caspase-7 may be a unique concomitant sensor of Ca^2+^ dysregulation, via changes in calpain-1 activity, and the classical caspase activation, two important death cascades in RGCs.

Thus, the objective of this study is to evaluate the involvement of caspase-7 in ON injury-induced RGC apoptosis in mice. In this study, injury to the ON was produced by intraorbital ON crush, which initiates rapid onset and progression of RGC apoptotic death [30, 31]. We showed that caspase-7, together with its upstream activator calpain-1, were activated and that the selective caspase-7 downstream substrates, P23 and kinectin, were hydrolyzed. We further showed that knockout of caspase-7 significantly both morphologically and functionally protected from the ON crush-induced loss of RGCs.

## Results

### Caspase-7 activation by ON injury

**Fig. 1:**
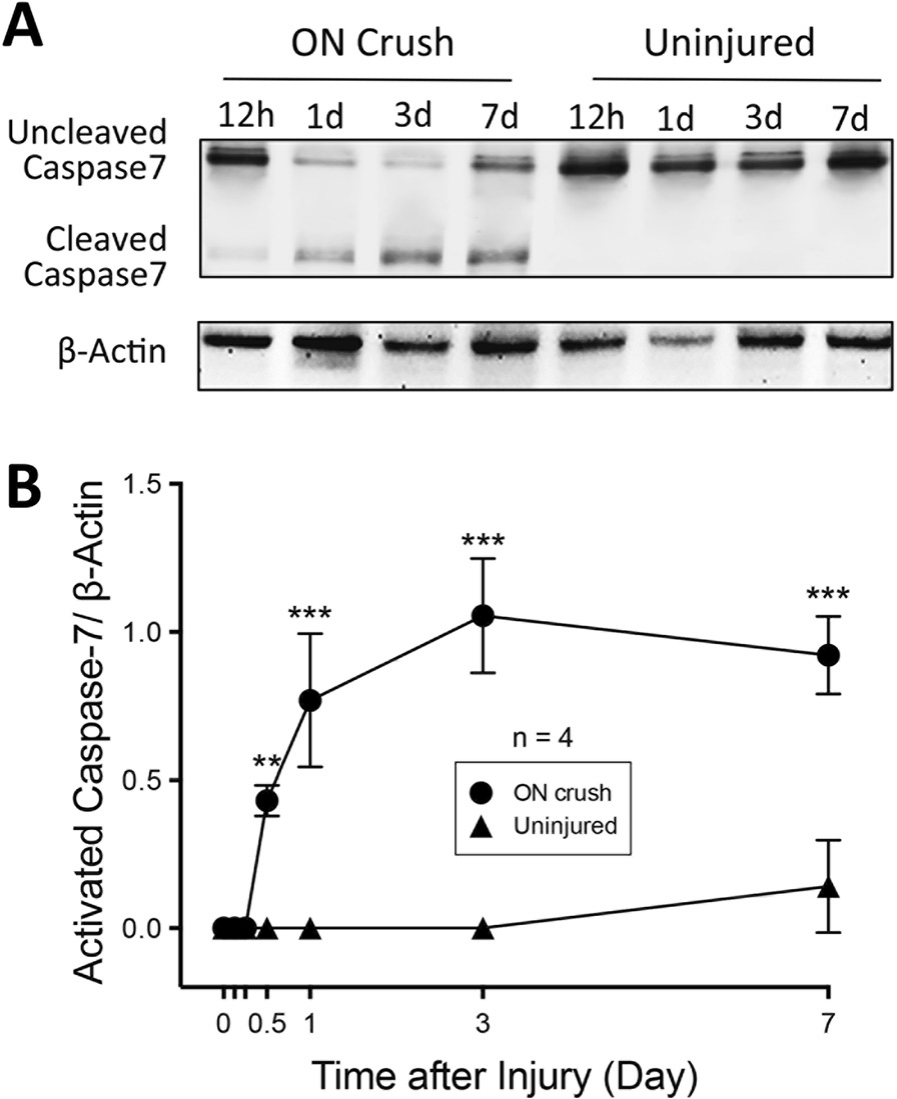
Activation of caspase-7 in mouse retina by ON crush. **a** Representative western blot images of pro- (*uncleaved*) and activated (*cleaved*) caspase-7 in protein extracts from ON crush (12 h, 1 d, 3 d, and 7 d post-injury) and control uninjured retinas of WT mice. **b** Densitometry analysis of western blot images showing a significant increase of active caspase-7 levels in ON crush eyes at the indicated time points when compared to uninjured eyes (*Right graph*). The values were normalized to β-actin and presented as the mean ± SD (*n* = 4). **: *p* < 0.01, ***: *p* < 0.001 by unpaired, two-tailed Student’s *t*-test

In control, uninjured mouse retinas, the level of activated (cleaved) caspase-7 was typically below the detection limit. Injury to the ON activated retinal caspase-7 in a time-dependent manner. Activated caspase-7 was not detected in western blots of retinal protein extracts of wild type (WT) mice at pre-injury, as well as 3 h and 6 h after ON crush. Active caspase-7 significantly (*p* < 0.01) increased at 12 h in ON crushed eyes compared to the uninjured control eyes. Levels continued to increase at 1 d and 3 d (*p* < 0.001), and appeared to plateau between 3 d and 7 d (Fig. 1), indicating that ON crush activated caspase-7 in the retina. It is interesting to note that while it takes 3 d [6] or later [32] for caspase-3 to be activated by ON injury, significant activation of caspase-7 in our study was observed at as early as 12 h after injury.

**Fig. 2:**
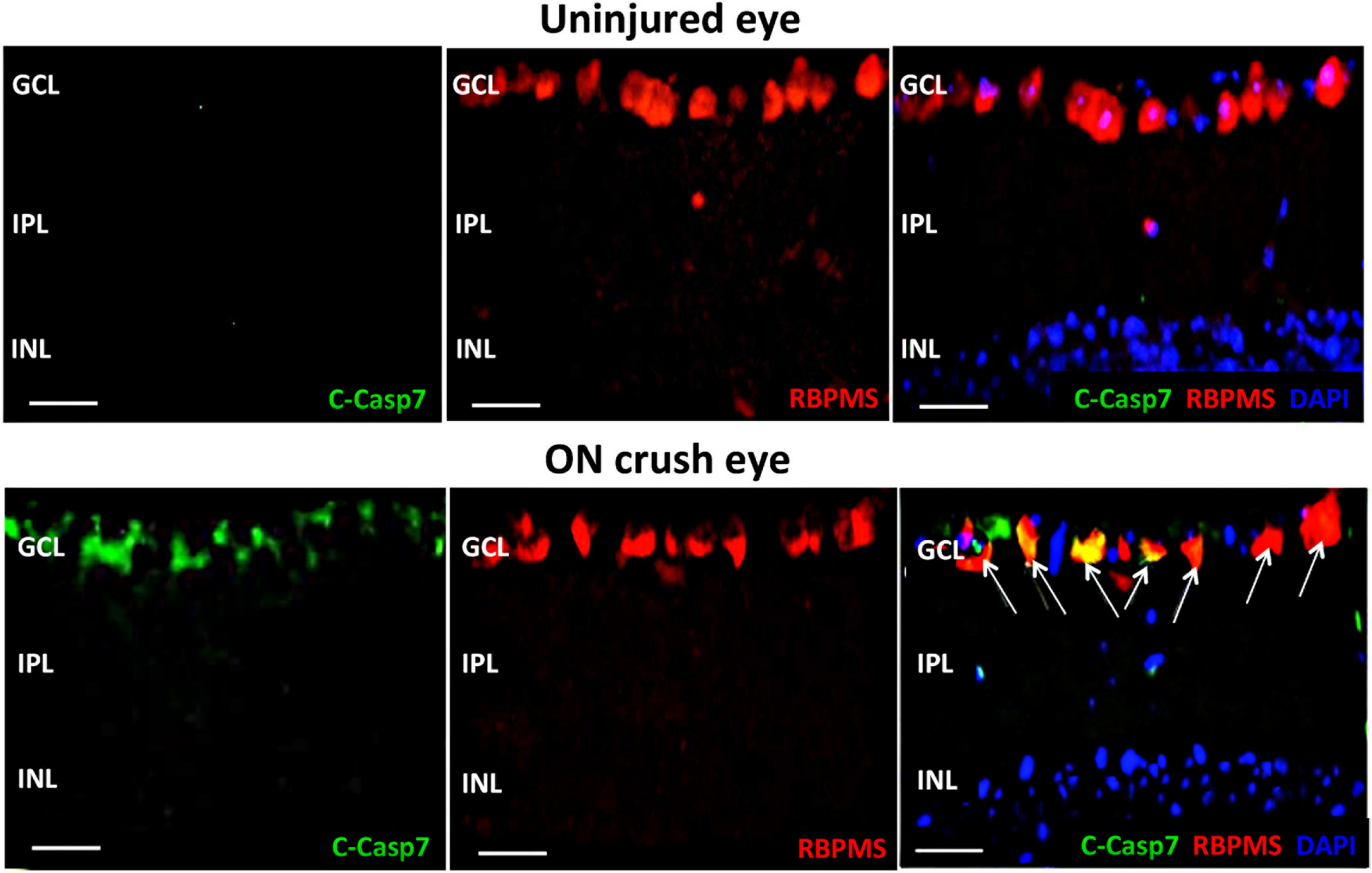
Activation of caspase-7 in RGCs by ON crush. Representative images of retinal cryosections from an uninjured control eye of WT mouse (*upper panel*) and an eye 3 d after ON crush (*lower panel*), immunolabeled with cleaved caspase-7 antibody (*green fluorescence*), RBPMS antibody (*red*), and DAPI (*blue*). ON crush increased cleaved caspase-7 levels mainly in the ganglion cell layer, most of which co-localized with the RGC-specific marker RBPMS (*arrows*). Scale bars = 50 μm. GCL: ganglion cell layer, IPL: Inner plexiform layer, INL: inner nuclear layer

The cellular localization of the activated caspase-7 in the retina was further evaluated by immunohistochemistry. Consistent with the Western blot analysis, there was no immunoreactivity of cleaved caspase-7 in the retina of uninjured eyes (Fig. 2). At 3 d after ON crush, there was an obvious increase in the level of cleaved caspase-7, which located mainly in the cytoplasm of cells in the ganglion cell layer (GCL) and co-localized with the specific RGC marker, RNA binding protein with multiple splicing (RBPMS) (Fig. 2), indicating that ON crush activates caspase-7 primarily in RGCs.

### Association of caspase-7 activation with calpain-1 activation

**Fig. 3:**
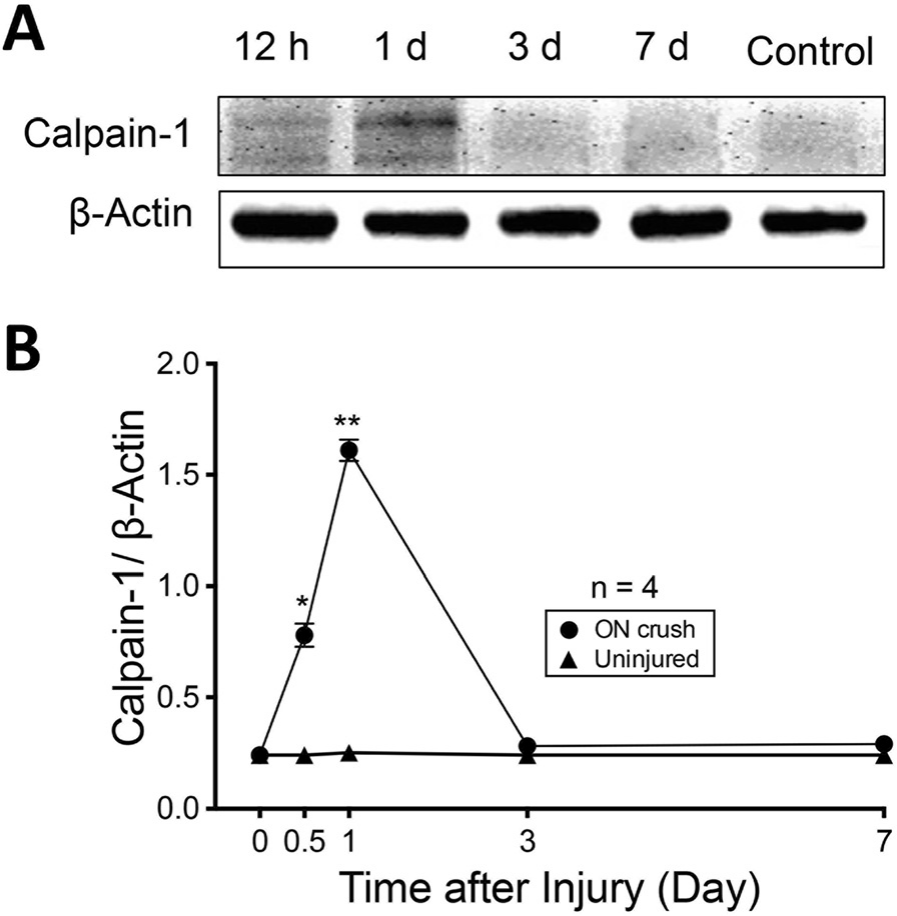
Activation of calpain-1 in mouse retina by ON crush. **a** Representative western blot images of calpain-1 in protein extracts after ON crush (12 h, 1 d, 3 d, and 7 d post-injury) and control uninjured retinas of WT mice. **b** Densitometry analysis of western blot images showing a significant increase of calpain-1 expression at 12 h and 1 d in ON crush eyes compared to uninjured eyes. The values were normalized to β-actin and presented as the mean ± SD (*n* = 4). *: *p* < 0.05, **: *p* < 0.01 by unpaired, two-tailed Student’s *t*-test

Calpain activation is associated with neuronal degeneration and cell death in a number of tissues, including the retina. In retinal abnormalities, the calpain pathway is involved in ischemia [33], excitotoxicity [34], experimental glaucoma [35], and photoreceptor degeneration [36, 37]. Caspase-7, unlike other caspases, is uniquely activated by calpain-1 [29]. Thus, we evaluated whether calpain-1 was involved in ON crush-induced RGC death. Western blot analysis demonstrated that calpain-1 was significantly activated in the retina at early time points of 12 h (*p* < 0.05) and 1 d (*p* < 0.01) after ON crush (Fig. 3). However, the level of activated calpain-1 returned to the basal level prior to 3 d. These findings are consistent with the notion that axonal damage induced a transient activation of calpain-1 that may trigger caspase-7 activation, which in turn contributes to the pro-apoptotic cascade in RGCs.

### Cleavage of caspase-7-selective substrates by ON injury

**Fig. 4:**
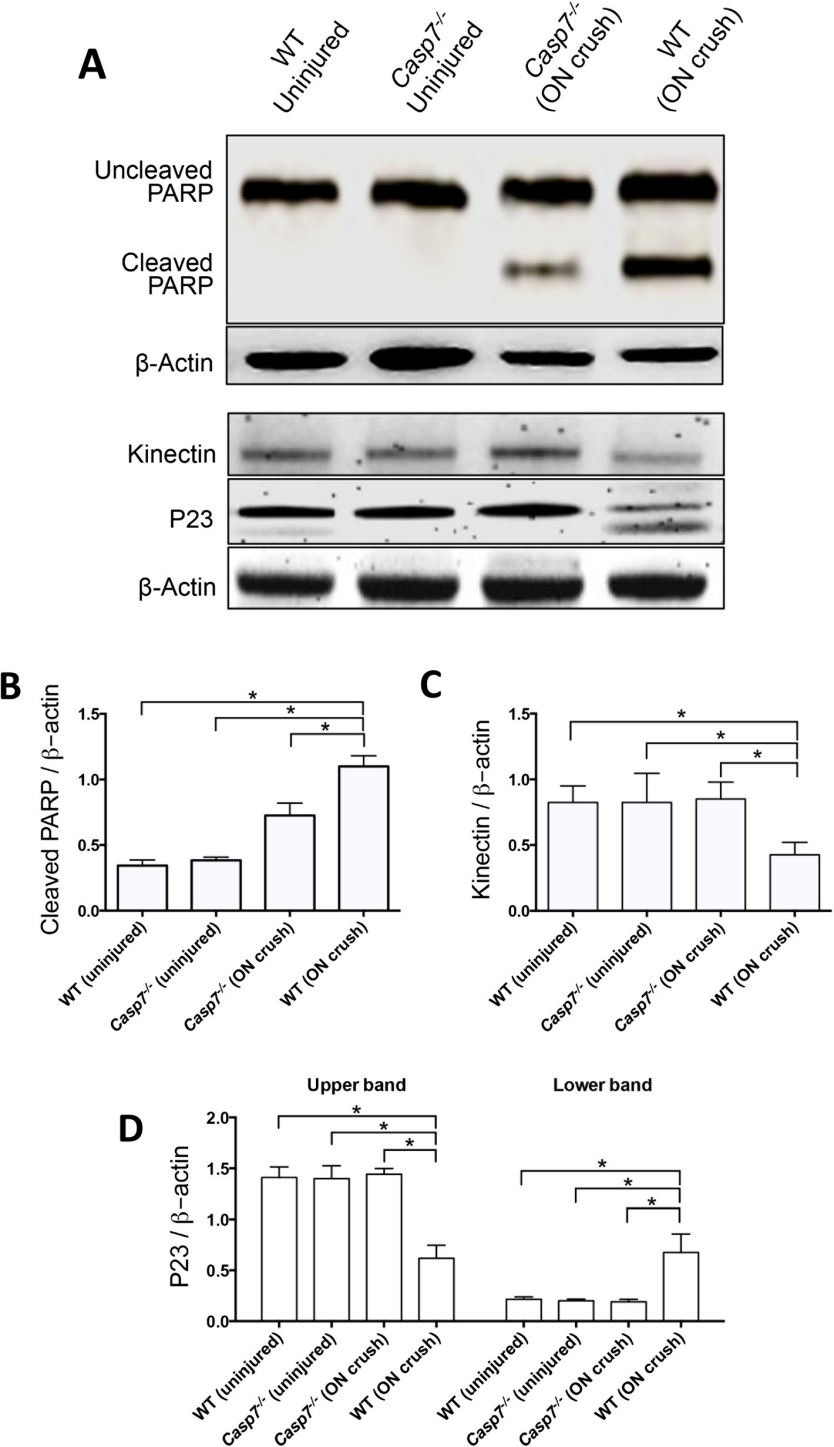
Cleavage of caspase-7 selective substrates by ON crush. **a** Representative western blot images of uncleaved and cleaved PARP, kinectin, as well as cleaved and uncleaved co-chaperone P23 in retinal protein extracts from WT or *Casp7* ^*−/−*^ mice, uninjured or 7 d after ON crush. **b**-**d** Densitometry analysis of the corresponding western blot images showing that ON crush induced hydrolysis of kinectin and P23 only in the WT mouse. The values were normalized to β-actin and presented as the mean ± SD (*n* = 4). *: *p* < 0.05 by One Way-ANOVA then Tukey–Kramer post hoc test

Effector caspases-3 and −7 facilitate apoptosis by hydrolyzing and activating downstream substrates. Some substrates, such as poly (ADP-ribose) polymerase (PARP), are common to both caspases. Other substrates, such as kinectin and co-chaperone P23, are cleaved selectively and more efficiently by caspase-7 compared to caspase-3 [23, 25, 24]. Therefore, we tested whether these caspase-7-selective substrates were cleaved in ON crush-induced retinal injury. To evaluate this, we compared protein extracts of whole retinas from ON crushed (7 d post injury) and uninjured eyes of WT and *Casp7*^*−/−*^ mice. We found minimal or undetectable hydrolysis of PARP, kinectin, or P23 in the uninjured retinas of either WT or *Casp7*^*−/−*^ mice (Fig. 4). Crush of the ON significantly (*p* < 0.05) increased hydrolysis of the non-selective substrate PARP in both mouse strains, while hydrolysis of caspase-7-selective substrates, kinectin and P23, was only observed in the WT but not the *Casp7*^*−/−*^ animals (Fig. 4). Collectively, these results indicate that ON crush activates caspase-7.

### Protection of *Casp7* knockout against ON crush-induced RGC loss

**Fig. 5:**
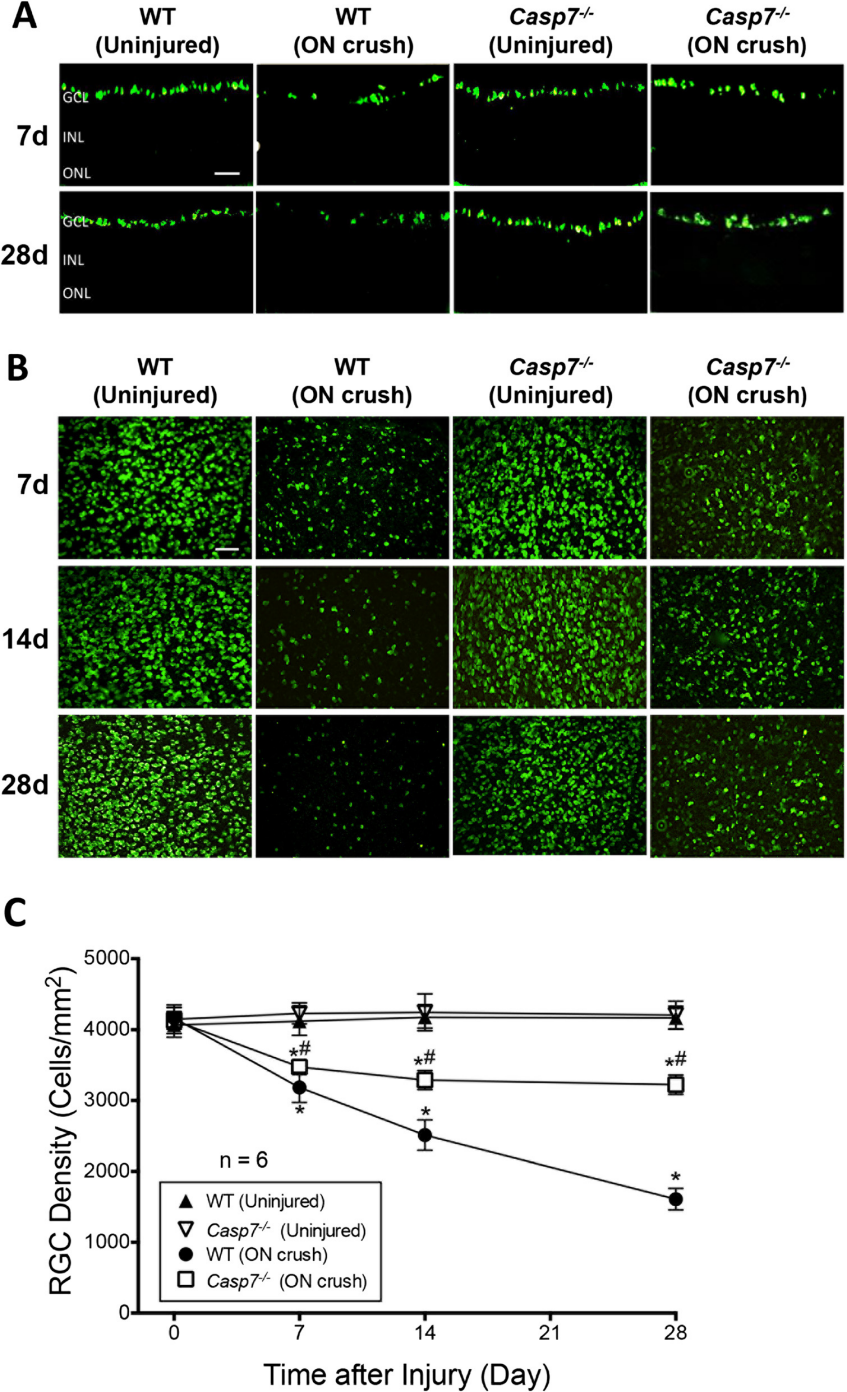
Protection by caspase-7 knockout against ON crush-induced RGC loss. **a** Representative retina cross-section images with RBPMS immunolabeling of WT and *Casp7* ^*−/−*^ mice. Images of both ON crush (7 d and 28 d post injury) and uninjured control eyes are shown. GCL: ganglion cell layer, INL: inner nuclear layer, ONL: outer nuclear layer. Scale bar = 100 μm. **b** Representative images showing RBPMS immunolabeled retinal flat-mounts of WT and *Casp7* ^*−/−*^ mice. Images of both ON crush (7 d, 14 d, and 28 d post injury) and uninjured control eyes are shown. Scale bar = 100 μm. **c** Quantitation of RBPMS-positive RGCs in uninjured and ON crush flat-mounted retinas of WT and *Casp7* ^*−/−*^ mice. There was no difference in RGC densities between uninjured eyes of WT and *Casp7* ^*−/−*^ mice. ON crush caused a steady decline in the number of RGCs in WT retina in comparison to *Casp7* ^*−/−*^ retina, which showed a significant preservation of RGCs. The values were represented as mean ± SD (*n* = 6). *: *p* < 0.05 versus the respective uninjured control group, #: *p* < 0.05 versus the “WT (ON crush)” group by One Way-ANOVA then Tukey–Kramer post hoc test

To assess if caspase-7 activation plays a critical role in ON injury-induced RGC apoptosis, we evaluated the effects of ON crush in *Casp7*^*−/−*^ mice compared to WT animals. The *Casp7*^*−/−*^ mouse has been characterized previously [38], and the knockout of caspase-7 protein expression was confirmed in this study (Additional file 1: Figure S1). As shown in retinal cross-sections (Fig. 5a), the densities of RBPMS-positive cells in the GCL of WT and *Casp7*^*−/−*^ mice were similar prior to ON crush. In the WT retinas, the RGC number declined both at 7 d and 28 d after ON crush, while there appeared less loss in the *Casp7*^*−/−*^ retina. To quantify this observation, RGCs were counted in RBPMS-labeled retinal flat mounts from each study group. Two 40x images were taken from peripheral and mid-peripheral regions of each of the four quadrants of each retina. Representative flat mount images from mid-peripheral regions are shown in Fig. 5b. The RGC numbers of the eight images from each retina were counted and averaged. Figure 5c demonstrates that RGC densities in uninjured eyes of WT and *Casp7*^*−/−*^ animals were similar and stable over the study period. ON crush caused a time-dependent loss of RGCs in WT mice. The loss was statistically significant (*p* < 0.05) at 7 d after injury, and continued to deteriorate at 14 d and 28 d. At 28 d, the remaining number of RGCs in the ON crush eyes was 38 ± 4 % (*n* = 6 retinas) that of the uninjured eyes. In contrast, although the number of RGCs in ON crush eyes of *Casp7*^*−/−*^ mice also significantly (*p* < 0.05) decreased starting from 7 d, the extent of RGC loss was significantly (*p* < 0.05) less in the *Casp7*^*−/−*^ mice compared to WT mice. Interestingly, the number of RGCs of these animals appeared to stabilize from 7 d to 28 d after ON injury and was statistically different from WT retinas at these time points. At 28 d, 76 ± 3 % of the RGCs remained, which was significantly (*p* < 0.05) higher than that of WT eyes. This finding indicates that knockout of caspase-7 partially protects the RGC loss induced by ON crush, and suggests that caspase-7 plays an important role in this pathophysiological change.

### Protection of *Casp7* knockout against ON crush-induced thinning of the retina

**Fig. 6:**
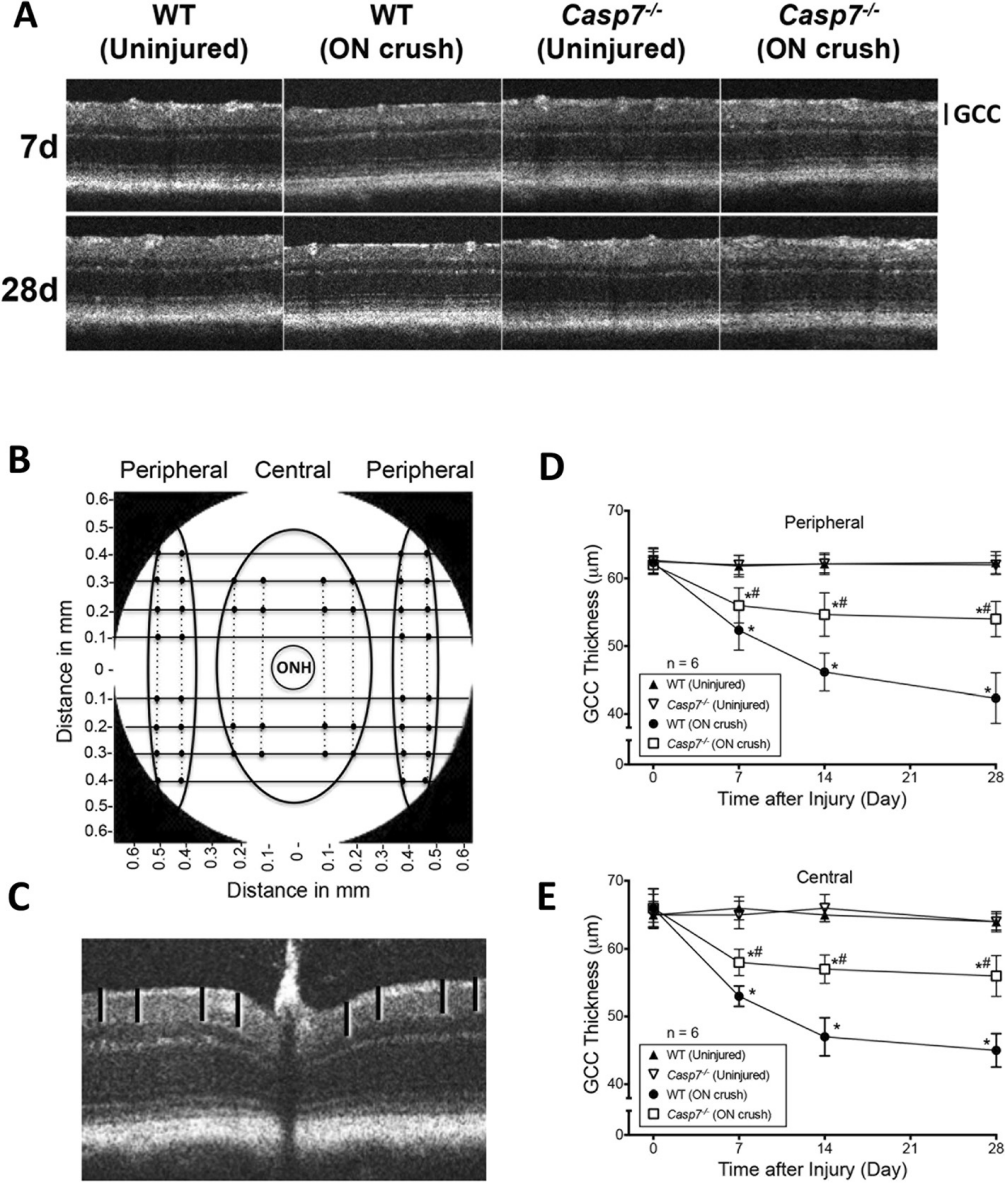
Protection by caspase-7 knockout against ON crush-induced thinning of retinal layers. **a** Representative SD-OCT images showing *in vivo* thickness of retinas of WT and *Casp7* ^*−/−*^ mice. Images of both ON crush (7 d and 28 d post injury) and uninjured control eyes are shown. GCC = combined thickness of NFL, GCL, and IPL. **b** Graphic representation of fundus of mouse eye showing approximate locations of retina thickness measurements. **c** Cross sectional images of mouse retina. Eight horizontal volume scans of retina at 100, 200, 300 and 400 μm through the area dorso-temporal from the ON (superior retina, 4 scans) and the area ventro-temporal from the ON (inferior retina, 4 scans) were used to evaluate GCC layer thickness. For measuring the GCC thickness at the peripheral retina, two calibrated calipers were placed at 400 μm and 500 μm on both sides of the center of each scan/optical slice. The peripheral thickness of the GCC was determined by averaging the 32 measurements. Similarly, to measure the GCC thickness of the central region of the retina, two calibrated calipers were placed at 100 μm and 200 μm at both sides of the center of each optical slice at 200 and 300 μm superior or inferior of the ON head. The central thickness of the GCC was determined by averaging the 16 measurements. Quantitation of GCC thickness in both the peripheral (**d**) and central (**e**) retinas of WT and *Casp7* ^*−/−*^ uninjured and ON injured eyes. The values were represented as mean ± SD (*n* = 6). *: *p* < 0.05 versus the respective uninjured control group, #: *p* < 0.05 versus the “WT (ON crush)” group by One-Way ANOVA then Tukey–Kramer post hoc test

In addition to the evaluation of *post-mortem* retinal tissues, we also used the spectral domain-optical coherence tomography (SD-OCT) to assess *in vivo* retinal layer thickness of WT and *Casp7*^*−/−*^ mice with or without ON crush. Our previous study showed that ON crush causes thinning of the retina, primarily due to thinning of the ganglion cell complex (GCC; comprising the nerve fiber layer (NFL), GCL, and inner plexiform layer (IPL)) [31]. In the current study, the GCC thicknesses at peripheral and central regions of the retina in uninjured eyes of WT and *Casp7*^*−/−*^ animals were equivalent and did not change over the 28 d study period (Fig. 6). Similar to our previous findings, thinning of GCC occurred at 7 d (*p* < 0.05) after injury in the ON crush eyes of WT mice. At 28 d, the thickness of GCC was only 68 ± 6 % and 70 ± 4 % of the uninjured eyes in peripheral and central retina, respectively. Most importantly, the ON crush-induced thinning of GCC was partially but significantly (*p* < 0.05) ameliorated in the *Casp7*^*−/−*^ mice (Fig. 6). At 28 d after injury, GCC thickness was 84 ± 4 % and 87 ± 5 % of that of uninjured eyes in peripheral and central retina respectively. These results provide temporal *in vivo* morphological evidence corroborating the protective effects of *Casp7* knockout against ON crush-induced RGC loss.

### Protection of *Casp7* knockout against ON crush-induced loss of RGC function

**Fig. 7:**
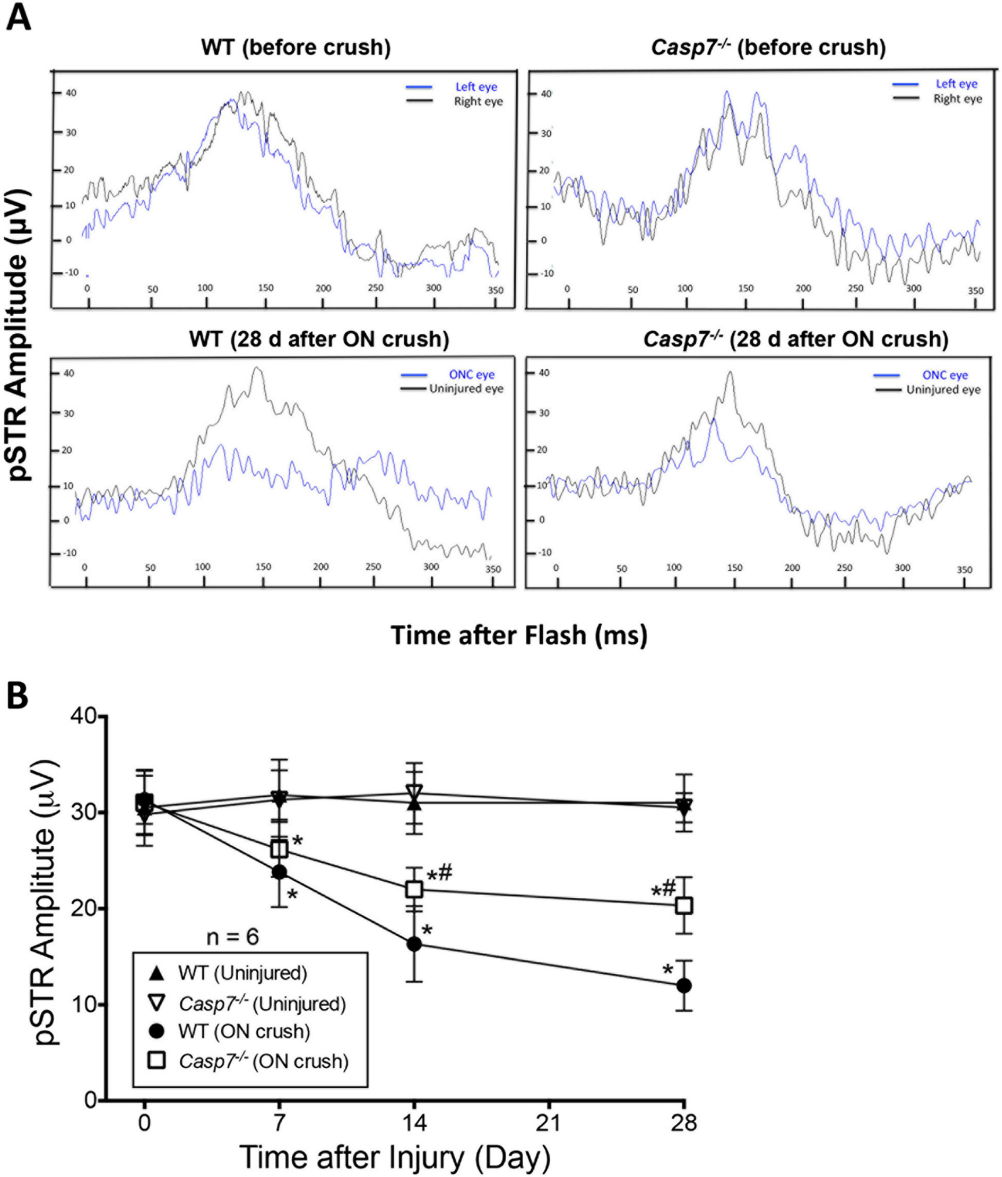
Protection by caspase-7 knockout against ON crush-induced loss of RGC function. **a** Representative STR-ERG waveforms from WT (*left panels*) and *Casp7* ^*−/−*^ (*right panels*) mice. Upper panels show the representative waveforms before ON crush and lower panels show the representative waveforms 28 d post crush. Blue lines represent the ON crushed left eyes and black lines represent the uninjured, contralateral (*right*) eyes. **b** pSTR amplitudes of ON crush and uninjured eyes of WT and *Casp7* ^*−/−*^ mice at indicated time points. ON crush significantly decreased RGC function in a time-dependent fashion as shown as a decline in pSTR amplitudes, which was significantly ameliorated in the *Casp7* ^*−/−*^ animals. Data are shown as mean ± SD (*n* = 6). *: *p* < 0.05 versus the respective uninjured control group, #: *p* < 0.05 versus the “WT (ON crush)” group by One-Way ANOVA then Tukey–Kramer post hoc test

In addition to morphological assessments, we also evaluated the potential protection of caspase-7 knockout against ON crush-induced loss of RGC function. The positive STR (pSTR) amplitude in the ERG has been shown to be a useful measurement of RGC function [31]. The pSTR amplitudes of uninjured eyes were similar between WT and *Casp7*^*−/−*^ mice (Fig. 7). ON crush induced a time-dependent, progressive, and significant (*p* < 0.05) decline in the RGC function in WT mice starting 7 d after injury. At 28 d, the pSTR was only 39 ± 8 % of that of uninjured eyes. RGC function was significantly (*p* < 0.05) protected by approximately half in the *Casp7*^*−/−*^ mice (Fig. 7). These results demonstrated that knockout of *Casp7* partially protected against the ON crush-induced RGC morphological and functional loss.

## Discussion

In many neurodegenerative diseases including glaucoma, axonal injury is a critical insult leading to apoptotic death of RGCs [39–41]. Although previous research has highlighted a central role of caspases as mediators of RGC apoptosis, the specific role of the distinct effector caspase, caspase-7, was not clear. We used ON crush as an experimental model of CNS injury to delineate this relationship. Similar to previous findings [31, 42, 43], we observed a ∼ 60 % decrease in RGC density at 28 d post injury in adult mice.

We found that caspase-7 was activated in RGCs following ON crush starting at 12 h post injury, and activation reached a plateau at 3 d. Consistent with this finding, we also showed the activation of calpain-1, an upstream activator of caspase-7, in the ON crush-injured retina. Since the enzymatic site of caspase-7 differs from that of caspase-3, substrates hydrolyzed by caspase-7 differ from those of caspase-3. In our study, we observed the hydrolysis of the caspase-7-selective substrates kinectin and co-chaperone P23. Together, our results demonstrate that ON crush activates caspase-7 in the mouse retina.

The finding that caspase-7 was activated in a majority of RGCs as early as 12 h after injury is intriguing. In comparison, activation of caspase-3 begins at 3 d [6] or later [32] after ON injury and is typically only observed in only a fraction of RGCs, likely those actively going through apoptosis. Since RGC death starts at or after 3 d post ON crush and not all RGCs immediately become apoptotic [31], our findings suggest that caspase-7 activation is involved in an earlier step in the apoptotic signaling cascade. Compared to other caspases, caspase-7 is unusual in that it can be activated by both the traditional initiator caspase pathway as well as a novel calpain-1 pathway. It is interesting to note that activation of calpain in our study was observed only at 12 and 24 h after ON crush injury, whereas, activation of caspase-7 was seen up to 7 days post injury. Together these data suggest that caspase-7 activation may be initiated by the calcium-calpain pathway and sustained by the traditional caspase activation cascade. The functional implication of this unique temporal profile and the exact role that caspase-7 plays require further investigation.

Although the exact role that caspase-7 activation in ON damage plays remains unclear, biological functions of substrates selectively hydrolyzed by caspase-7 may provide some clues. Co-chaperone P23, which was hydrolyzed in the injured retina of only WT but not *Casp7*^*−/−*^ mice, is a ubiquitous protein found in most eukaryote tissues. It binds and stabilizes Heat-shock protein 90 kDa (Hsp90), serves as an essential component of the Hsp90 molecular chaperone complex [44], and interacts with a variety of Hsp90 clients, including most steroid receptors, active telomerase, tyrosine kinases, and transcription factors [45]. In addition, genomic and proteomic studies further revealed an extensive P23 molecular network independent of Hsp90 clients [46]. Most intriguingly, this protein is recognized as a pro-survival factor in cancer cells [25] and perinatal mice [47]. Thus, caspase-7 mediated hydrolysis of P23 will disrupt these intricate biological interactions and may synergize with the canonical caspase-mediated cell death pathway. Kinectin, another caspase-7 selective substrate, is found on the cytoplasmic face of endoplasmic reticulum (ER) membranes [48], as well as the cargo-binding site of conventional kinesins. It has been implicated in the formation, stabilization, shaping, and cytoskeletal interactions of sheet-like cisterns of the ER [49]. As a motor-binding protein, kinectin is also associated with active intracellular organelle movements powered by kinesin, thus controlling intracellular cargo transport [50]. These vital functions of kinectin are expected to be compromised by caspase-7 activation.

To investigate the importance of caspase-7 in ON crush-induced RGC loss, we assessed the potential RGC protective effects of *Casp7* knockout. Our results showed significant protection of RGCs in the ON crushed eyes of *Casp7*^*−/−*^ mice when compared to WT mice 7 to 28 d post crush. We demonstrated this protection using both morphological and functional evaluations. Morphologically, we analyzed *post mortem* RGC density and measured *in vivo* thickness of retinal layers. RGC counts of flat-mounted retinas indicated that ON crush dramatically reduced the density of these neurons in WT mice, with only 38 % remaining at 28 d post injury. In contrast, 76 % of the RGCs survived this insult in *Casp7*^*−/−*^ mice. An interesting observation is that RGC density of these animals appeared to stabilize from 7 d to 28 d compared to the continuous decline in the WT animals. This suggests that the protective effect of *Casp7* knockout is not a transient delay of neuronal death, and instead it may provide a long-term protection.

SD-OCT analysis allows non-invasive, *in vivo* monitoring of morphological changes of the retina. We showed that GCC thickness significantly decreased in WT mice from 7 to 28 d post ON crush. GCC is comprised of three retinal layers: the NFL, containing RGC axons; the GCL, containing RGC soma; and the IPL, containing the RGC dendrites. ON injury causes loss of RGCs and thinning of the GCC [51–53]. In comparison, ON crush-induced thinning of the GCC was significantly and stably ameliorated in *Casp7*^*−/−*^ mice from 7 to 28 d after injury. Together, the *in vivo* and *post mortem* results indicate that absence of caspase-7 protects against morphological deterioration of RGCs caused by ON injury.

Most importantly, knockout of *Casp7* not only preserved RGC density and structures, but also significantly protected against loss of RGC function subsequent to ON crush. The STR response is derived mainly from RGCs [54]. In WT mice, we found an approximately 35 % decrease in pSTR responses at 7 d and approximately 65 % at 28 d after ON crush. This coincides with a time-dependent RGC loss in WT mice after ON crush. In contrast, the pSTR response in *Casp7*^*−/−*^ mice showed only a 15 % reduction at 7 d and a 25 % decrease at 28 d after ON crush, clearly showing protection of RGC function.

Our discovery that caspase-7 plays a critical role in ON crush-induced RGC loss is important for the following reasons. Being a concomitant sensor of both the calpain and traditional caspase pathways, caspase-7, through hydrolysis of its specific downstream substrates may regulate unique and yet-to-be-revealed mechanisms in controlling cell death. In addition, caspase-7 is a p53 responsive gene; p53 binds to the first intron of the human and mouse caspase-7 gene [55]. p53 is a transcription factor, involved in the regulation of neuronal viability after injury [56, 57]. Activated p53 can modulate mitochondrial membrane permeability and the release of mitochondrial proteins during apoptosis. p53 is phosphorylated and its pro-apoptotic targets PUMA and Fas/CD95 are upregulated in ON axotomy-induced RGC death [58]. It is interesting to speculate that caspase-7 may be involved in this apoptosis signaling mechanism.

Based on morphological and functional criteria, we showed that *Casp7* knockout was protective against ON crush-induced loss of RGCs. However, this protection was not complete (i.e., it did not reach the uninjured levels), suggesting that caspase-7 is not the sole mediator of this ON crush pathology and that other cellular pathways may be involved. It is likely that caspase-3 also contributes to this injury. This is consistent with the findings that inhibition of caspase-2, an upstream initiator caspase for both caspases-3 and −7, via pharmacological inhibition or siRNA provided almost complete protection of RGC against ON crush-induced death for at least 30 days [5, 21]. Furthermore, caspase-independent pathways may also be involved in neuronal cell death associated with ON injury, such as mitochondria-mediated death. Mitochondria are essential in controlling cell death. Exposure of RGCs to TNF-α or hypoxia leads to mitochondrial alterations, including the release of cytochrome c and apoptosis inducing factor, leading to RGC death through caspase-independent events [59]. After ON injury, amplification of TNF-α activates JNK, which causes RGC death [60]. Fernandes et al. have shown that JNK signaling is a major early pathway triggering RGC death after axonal injury [61]. Moreover, reactive oxygen species are associated with mitochondrial dysfunction and may be involved in signaling RGC apoptosis after axonal injury [62]. Therefore, it is clearly important to delineate the relative roles each of these signaling pathways plays and their interactions in ON crush-induced RGC death and other forms of CNS injury.

## Conclusions

In conclusion, our findings demonstrated the important role of caspase-7 in RGC apoptosis induced by ON crush. Our results also show that knockout of *Casp7* partially protects against ON crush-induced RGC loss and the thinning of inner retinal layer thickness. Most importantly, we found that RGC function was protected by *Casp7* knockout. Thus, inhibition of caspase-7 activity may be a novel therapeutic strategy for neurodegenerative diseases of the retina and the ON. Further studies need to be performed to unravel the full mechanisms of caspase-7-dependent and independent apoptotic pathways in ON injury induced RGC apoptosis.

## Methods

### Animals

Female C57BL/6 J mice, serving as the WT control, and caspase-7 knockout (*Casp7*^*−/−*^) (B6.129S6-*Casp7tm1Flv*/J) breeders were purchased from the Jackson Laboratory (Bar Harbor, ME). The *Casp7*^*−/−*^ mice were backcrossed with the C57BL/6 J strain for at least 10 generations, making the C57BL/6 J strain an appropriate WT control. All mice were maintained in 12:12 light/dark cycle and supplied with food and water *ad libitum*. All experiments were conducted in accordance with the ARVO Statement of the Use of Animals in Ophthalmic and Vision Research and the University of North Texas Health Science Center’s Institutional Animal Care and Use Committee guidelines.

### Optic nerve crush

ON crush was performed on the left eyes of C57BL/6 J and *Casp7*^*−/−*^ mice at 8–10 weeks of age according to a previously described procedure [31]. Briefly, mice were anesthetized by intraperitoneal injection of ketamine and xylazine, (100 and 10 mg/kg body weight, respectively). The cornea was anesthetized with a topical drop of 0.5 % proparacaine HCl (Alcon Laboratories, Fort Worth, TX). The ON of the left eye was exposed intraorbitally through a small opening made among the surrounding extraocular muscles. A pair of self-closing forceps was used to crush the ON approximately 1 mm behind the globe for 4 s. Care was taken not to block the blood flow to the retina through the central retinal artery. The eye was covered with antibiotic ointment (polymyxin B sulfate and bacitracin; Bausch & Lomb, Tampa, FL). All animals undergoing surgery were given buprenorphine (0.05 mg/kg, subcutaneously) immediately after surgery.

### Western blot analysis

Retinal protein extracts were obtained from dissected retinas by sonication in mammalian protein extraction buffer (Thermo Scientific, Rockford, IL) containing protease inhibitor and phosphatase inhibitor cocktails (Thermo Scientific). Protein concentrations were determined using the Bio-Rad Dc protein assay according to the manufacturer’s instructions (Bio-Rad Laboratories, Richmond, CA). A total of 60 μg protein from each sample was loaded and electrophoresed on 15 % denaturing polyacrylamide gels and then electrophoretically transferred to polyvinylidene fluoride membranes. Blots were incubated in 5 % milk in Tris Buffered Saline Tween solution (TBST; 20 mM Tris, 0.5 M NaCl, and 0.05 % Tween 20, pH 7.4) for 1 h to block nonspecific binding. Membranes were incubated overnight at 4 °C with primary antibodies diluted in Super Block T20 (Thermo Scientific). The primary antibodies used were caspase-7 (1:1000, Cell Signaling, Danvers, MA, Cat # 9492), calpain-1 (1:1000, Cell Signaling, Cat # 2556), kinectin (1:1000, Novus Biologicals, Littleton, CO, Cat # NBP1-91592), P23 (1:1000, GeneTex, Irvine, CA, Cat # GTX112655), PARP (1:1000, Cell Signaling, Cat # 9542), and mouse-anti-β-actin (1:5000; clone C4, Millipore, Billerica, MA, Cat # MAB1501). The following day, membranes were washed with TBST 3X for a total of 30 min and were incubated for 1 h with corresponding secondary antibodies: goat-anti-rabbit-horseradish peroxidase (1:10,000; Thermo Scientific, Cat # 32460) or goat-anti-mouse-horseradish peroxidase conjugated antibodies (1:10,000; Thermo Scientific, Cat # 32430) diluted in TBST. Membranes were washed 3X with TBST for 10 min each at room temperature. The proteins were then visualized in a FluorChem 8900 imager (Alpha Innotech, San Leandro, CA) using ECL detection reagent Super Signal West Femto Maximum Sensitivity Substrate (Thermo Scientific). The relative expression of proteins was determined by densitometry analysis and then normalized to β-actin.

### Immunohistochemistry

Eyes from WT and *Casp7*^*−/−*^ mice were enucleated and fixed overnight in freshly prepared 4 % paraformaldehyde (PFA) in phosphate-buffered saline (PBS). Afterwards, eyes were submerged sequentially in solutions of 10 %, 20 % and 30 % sucrose for at least 1 h each. Eyes were embedded in cryostat compound (Tissue TEK OCT, Sakura Finetek, Torrance, CA) and frozen at −80 °C. Sections (10 μm thick) were cut using a cryostat (Leica Microsystem, Buffalo Grove, IL) and transferred to glass slides. Retinal cryosections were soaked in PBS for 10 min and blocked in 2 % goat serum, 0.3 % Triton X-100 in PBS for 1 h at room temperature. The sections were double immunolabeled with mouse monoclonal caspase-7 antibody (1:50, Santacruz Biotechnology, Dallas, TX, Cat # SC-28295) and rabbit polyclonal RBPMS antibody (1:200, Genetex, Irvine, CA Cat # GTX118619) overnight at 4 °C, washed three times with PBS and incubated for 1 h with the respective secondary antibodies at room temperature. Secondary antibodies used were AlexaFluor488 goat-anti-rabbit (1:1000; Life Technologies, Grand Island, NY, Cat # A11008) and tetramethylrhodamine goat-anti-mouse IgG (1:1000; Life Technologies, cat # T2762). Sections were mounted with Vectashield Mounting Medium containing DAPI (Vector Laboratories, Burlingame, CA). Images were acquired using a Carl Zeiss inverted microscope Axiom vision series (Carl Zeiss Microscopy, Thornwood NY).

### Retinal flat mounts and cell counts

Following fixation in 4 % PFA, the anterior segment of each eye was removed and the posterior eyecup was processed for whole mount immunostaining to quantitate RGCs. Retinas from fixed eyes were carefully dissected, flat-mounted on glass slides, and blocked in 0.3 % Triton X-100 in PBS containing 2 % goat serum for 2 h. Retinas were then incubated in rabbit polyclonal RBPMS antibody (1:200, diluted in 0.3 % Triton X-100 in PBS) overnight at 4 °C. Following washes in PBS, the retinas were incubated with AlexaFluor488 goat-anti-rabbit (1:1000, diluted in PBST) for 24 h at 4 °C and then mounted with Vectashield Mounting Medium containing DAPI (Vector Laboratories). Eight 40x images were taken from peripheral and mid-peripheral regions of the four quadrants of each retina. The number of labeled cells from each image was counted. For each individual retina, the RGC count was obtained by averaging the eight counts for each retina.

### Spectral domain-optical coherence tomography

SD-OCT imaging was performed using the Bioptigen Envisu™ Ophthalmic Imaging System (Bioptigen, Durham, NC) to measure GCC thickness (combination of NFL, GCL, and IPL layers). Mice were anesthetized using a ketamine/xylazine cocktail (100/10 mg/kg), and their pupils were dilated with a drop of 2.5 % phenylephrine hydrochloride (Bausch & Lomb). To prevent corneal desiccation during the procedure, topical Systane Ultra® lubricant eye drop (Alcon) was applied frequently and bilaterally. The retina was scanned with the rectangular scanning mode consisting of 100 B-scan tomograms, each with 1000 A-scans, by using InVivoVue software (Bioptigen Inc. Durham, NC). Horizontal volume scans of retina at 100, 200, 300 and 400 μm through the area dorso-temporal of the ON head (superior retina) and the area ventro-temporal of the ON head (inferior retina) were used to evaluate the thickness of the GCC. For measuring the GCC thickness at peripheral retina, two calibrated calipers were placed at 400 μm and 500 μm on both sides from the center of each of the eight optical slices. The peripheral GCC thickness of each retina was determined by averaging the 32 measurements. To determine GCC thickness at central retina, two calibrated calipers were placed at 100 μm and 200 μm on both sides of the center of optical slices that were 200 and 300 μm superior or inferior to the ON head. The central thickness of the GCC of each retina was determined by averaging the 16 measurements.

### Scotopic threshold response electroretinography

The STR of ERG was performed with the Handheld Multi-species-ElectroRetinoGraph unit (Ocuscience, Kansas City, MO) to assess the functional analysis of RGCs [31]. Mice were dark-adapted overnight prior to ERG recordings. During the whole procedure, mice were anesthetized using 1.5 % isoflurane. The mouse pupils were dilated with a drop of 2.5 % phenylephrine, and the cornea was anesthetized with a drop of 0.5 % proparacaine. A ground electrode was inserted subcutaneously in the tail and two reference electrodes were inserted subcutaneously underneath both eyes. Recording electrodes were placed on the cornea. To maintain moisture, a drop of 2.5 % methylcellulose was added to the concave side of a contact lens, which was placed on the electrodes. Light stimuli (0.03 mcd · s/m^2^) were generated in a Ganzfeld dome with light emitting diodes. Responses from 10 flashes with 2 s intervals were averaged. The positive component of the STR amplitude was quantified and analyzed by using ERGVIEW software (Ocuscience).

### Statistical analysis

Results between two groups were analyzed for differences using the unpaired Student’s *t*-test (GraphPad Prism 6.0; GraphPad Software, La Jolla, CA). Results among three or more groups were compared using one-way ANOVA followed by Tukey–Kramer post hoc test. Statistical significance was determined at p values < 0.05.

## Additional file

Additional file 1: Figure S1.
Reduction in caspase-7 expression in the retina of *Casp7* ^*−/−*^ mice. Representative western blot images of pro-caspase-7 in protein extracts from retinas from WT and *Casp7* ^*−/−*^ mice. (TIFF 42 kb)

## Abbreviations

*Casp7*^*−/−*^: Caspase-7 knockout
CNS: Central nervous system
ER: Endoplasmic reticulum
GCC: Ganglion cell complex
GCL: Ganglion cell layer
Hsp90: Heat-shock protein 90 kDa
INL: Inner nuclear layer
IPL: Inner plexiform layer
NFL: Nerve fiber layer
ON: Optic nerve
PARP: Poly (ADP-ribose) polymerase
pSTR: Positive component of the scotopic threshold response
RBPMS: RNA-binding protein with multiple splicing
RGC: Retinal ganglion cell
SD-OCT: Spectral domain-optical coherence tomography
WT: Wild type
